# Trends and Research Hotspots in Biomarkers of Sjögren's Syndrome Over the Past Two Decades: A Data‐Driven Atlas Analysis

**DOI:** 10.1002/iid3.70462

**Published:** 2026-05-06

**Authors:** Xinyan Zhang, Huan Li, Xueqing Zhou, Shangwen Qi, Songwei Li

**Affiliations:** ^1^ The First Clinical Medical College Henan University of Chinese Medicine Zhengzhou Henan China; ^2^ Department of Rheumatology and Immunology The First Affiliated Hospital of Henan University of Chinese Medicine Zhengzhou Henan China

**Keywords:** bibliometrics, biomarkers, hotspot analysis, Sjögren's syndrome

## Abstract

**Background:**

Sjögren's syndrome (SS) is a chronic, systemic inflammatory disorder primarily characterized by dry eyes and dry mouth, often involving multiple organ systems. The disease's heterogeneous clinical presentation and the absence of non‐invasive, specific biomarkers complicate its diagnosis and prognostic assessment.

**Methods:**

Literature was sourced from the Web of Science Core Collection, and a bibliometric network was constructed using VOSviewer, CiteSpace, and Bibliometrix. This analysis covered countries/regions, institutions, journals, authors, citations, and keywords, offering an overview of SS biomarker research and identifying future directions.

**Results:**

A total of 1118 articles on SS biomarkers were analyzed. Publication trends fluctuated over the years, with China and the United States leading in volume and citation frequency. The Assistance Publique‐Hôpitaux de Paris was the most prolific institution. Among researchers, Xavier Mariette dominated the field, with leading positions in volume of publications, h‐index, and total citations. *Annals of the Rheumatic Diseases* emerged as the most influential journal. Over the past two decades, research has expanded significantly, with emerging themes including “DNA methylation,” “genes,” “interstitial lung disease,” and “data‐driven,” indicating future focus areas such as epigenetics, severe complication biomarkers, and multi‐omics studies.

**Conclusion:**

This bibliometric analysis provides a comprehensive view of SS biomarker research, highlighting recent trends and future research directions, offering valuable insights for ongoing studies.

## 1 Introduction

Sjögren's syndrome (SS) is a systemic autoimmune disorder characterized by lymphocytic infiltration of exocrine glands such as the salivary and lacrimal glands. The global prevalence of this disease exhibits significant regional variation, with reports ranging from 0.29% to 0.77% in China, while certain European countries report a prevalence of approximately 0.23% [1]. The exact pathogenesis of SS remains incompletely understood, involving a complex interplay between genetic susceptibility, environmental factors, immune dysregulation, and the interaction of multiple cytokines. This uncertainty surrounding the disease mechanism has heightened the complexity of identifying therapeutic targets. Furthermore, SS is marked by considerable clinical heterogeneity, with substantial variation in symptom severity among patients. In addition to the characteristic dry mouth and dry eyes, approximately 30%–50% of patients may develop systemic complications, such as lymphoma, interstitial lung disease, and neurodegenerative conditions, leading to multi‐organ involvement and significantly affecting life expectancy [2]. Although personalized treatment is theoretically the optimal strategy, the lack of refined classification criteria has severely hindered the implementation of precision medicine in clinical practice.

The unpredictability of disease progression, coupled with the absence of reliable predictive measures, presents substantial challenges in clinical management. Currently, the diagnosis and disease monitoring of SS rely primarily on a comprehensive evaluation of clinical symptoms, laboratory markers, imaging findings, and tissue histopathological examination. Among these, lip gland biopsy results and positive serum anti‐SSA/Ro antibodies are considered key diagnostic indicators [3]. However, existing biomarkers suffer from insufficient sensitivity and limited discriminatory power across different patient subgroups [4]. Additionally, lip gland biopsy, being an invasive procedure, carries inherent risks, and its pathological interpretation is susceptible to inter‐observer variability [5]. These diagnostic limitations underscore the urgent need for novel biomarkers. With the advancement of our understanding of SS pathogenesis and the standardization of biomarker concepts, several potential new markers have been identified. Nevertheless, there remains a significant gap in comprehensive, visualized analyses of the evolution and development trends of SS‐related biomarkers.

Bibliometrics, a research methodology that employs mathematical and statistical techniques to quantitatively analyze scientific literature, focuses on the evaluation of publication and citation data [6]. In this study, we utilize CiteSpace (6.2.R3), VOSviewer (v1.6.20), R‐bibliometrix (v4.3.3), and Microsoft Excel (2019) to analyze research publications related to SS biomarkers from 2004 to 2025. This study explores current research hotspots in the field and offers insights into future research directions.

## 2 Materials and Methods

### 2.1 Data Sources

**Figure 1:**
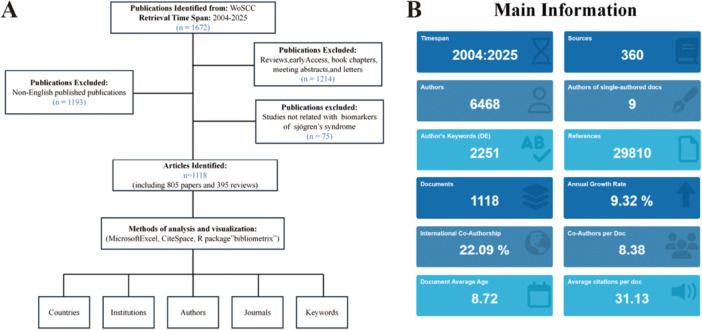
(A) Flowchart of the literature search and selection process. (B) Overview of the annual publication volume of biomarkers of SS from 2002 to 2025.

The data for this study were sourced from the Web of Science Core Collection (WoSCC) database, a globally recognized authoritative citation database. An exact search strategy was employed for literature retrieval, with the search query formulated as follows: (TS = (biomarker* OR marker* OR “Surrogate Endpoints” OR “Surrogate End Points” OR “Surrogate Endpoint” OR “Surrogate End Point”)) AND TS = (“Sjögren's syndrome” OR “sjogren syndrome” OR “sicca syndrome”). The search covered the period from January 2004 to December 2025, with data retrieval performed on March 6, 2026. All searches were conducted within a single day. Only “Articles” were included in the final dataset, while “Review Articles”, “Early Access”, “Proceedings Papers”, and “Book Chapters” were excluded. This approach ensures that the identified hotspots and co‐citation networks reflect foundational primary studies and actual research activity within the field of SS biomarkers. Due to limitations in the analysis software, articles not published in English were also excluded, yielding a total of 1672 articles. Two independent researchers reviewed the titles and abstracts of the retrieved articles to extract relevant data and conduct a comprehensive evaluation and selection. Any discrepancies between the two researchers were resolved through discussion with a third researcher to ensure the reliability and validity of the study. Ultimately, 1118 articles were included, and the retrieval results were exported as “Full Record and References” and saved as a plain text file for further processing (Figure 1A).

### 2.2 Analysis Methods

For data analysis, CiteSpace (version 6.2.R3), VOSviewer (version 1.6.20), and the R package “bibliometrix” (version 4.4.2) were employed to conduct a visual analysis of the global research trends in SS‐targeted therapy through multidimensional data mining and network relationship construction. CiteSpace, with its core function of constructing scientific knowledge maps, excels in displaying the dynamic evolutionary characteristics of research fields [7]. VOSviewer specializes in intuitive network mapping and density visualization, making it particularly suitable for collaboration and co‐occurrence analysis [8]. As an open‐source R package, “Bibliometrix” offers comprehensive support for the entire process, from data cleaning to statistical modeling [9]. To ensure consistency in the benchmarking, the results of these three tools were compared using WoSCC data from 2004 to 2025.

## 3 Results

### 3.1 Global Overview

To analyze a particular discipline or field, it is essential to first clarify the overall status of the related literature, such as the time span, volume of publications, and number of contributing authors. A total of 1118 research articles related to biomarkers in Sjögren's syndrome (SS) from 2004 to 2025 were collected. These articles were authored by 6468 researchers from 46 countries, with only 9 independent researchers identified. The international collaboration rate stood at 22.09%, with an average of 8.13 authors per article, highlighting the pivotal role of global, multi‐center cooperation in advancing the field. The core literature had a high impact, with an average of 31.13 citations per article, underscoring the academic value of this research direction. The average age of the included articles was 8.72 years, indicating that research published in the last 9 years constitutes the core of the current knowledge system (Figure 1B).

### 3.2 Trends in Publication Volume

**Figure 2:**
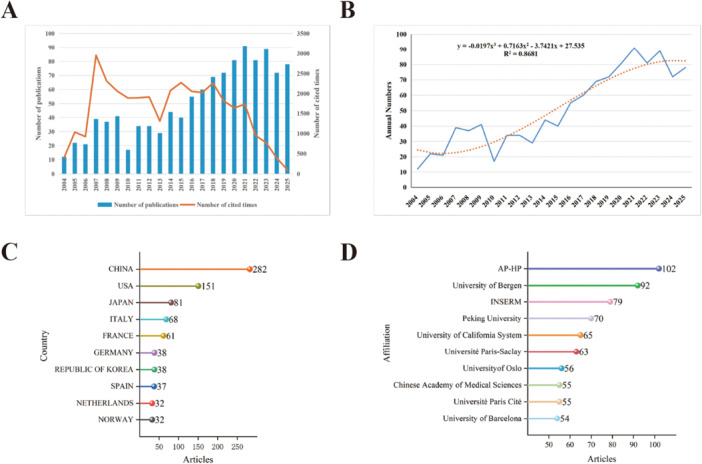
(A) Global trend of annual publications and citations related to biomarkers of SS from 2004 to 2025. (B) The fitting curve of the annual publication volume. (C) The top 10 countries/regions contributing to biomarkers of SS. (D) The top 10 institutions with the most publications contributing to biomarkers of SS.

A direct method of assessing the rise and fall of a research topic is through its annual publication trends. The annual growth rate for publications in this field was 9.32%, surpassing the global average growth rate for scientific output (Figure 1B). The trends in publication volume and citation frequency from 2004 to 2025 were subsequently analyzed. From 2004 to 2013, the volume of publications remained at a relatively low and fluctuating level, with an average of 28.6 articles per year. From 2014 to 2021, a steady increase in publications was observed, reaching the peak of 91 articles in 2021. After 2022, publication volume showed slight fluctuations but remained at a high level. Additionally, the total citation frequency of the included articles peaked in 2007. Despite the high volume of publications after 2019, total citations have shown a downward trend (Figure 2A). The polynomial fit curve for the annual trend reveals the nonlinear growth nature of the publication volume (Figure 2B). The R² value of 0.8681 indicates that the model explains approximately 85% of the variation, with the remaining 15% likely influenced by external factors such as policy changes and technological breakthroughs. Overall, these data suggest that research on biomarkers in SS is still progressing rapidly and continues to garner significant academic attention.

In the global landscape of SS biomarker research, China (282 articles) demonstrates an absolute leading edge. This reflects the dominant positions in terms of foundational research resources, clinical sample size, and research investment. Following closely are USA (151 articles), Japan (81 articles), Italy (68 articles), and France (61 articles), with Germany (38 articles), Republic of Korea (38 articles), Spain (37 articles), the Netherlands (32 articles), Norway (32 articles) and rounding out the top contributors (Figure 2C). In terms of institutional publication volume, the Assistance Publique‐Hôpitaux de Paris (AP‐HP) ranked first globally. This is followed by the University of Bergen with 92 articles and Institut National de la Santé et de la Recherche Médicale (INSERM) with 79 articles, representing a strong European leadership in this domain. In the Asian region, Peking University led the research output with 70 articles, followed by the Chinese Academy of Medical Sciences and Université Paris Cité, both tied with 55 articles. The University of California System represented the primary American contribution with 65 publications. Other notable institutions include Université Paris‐Saclay (France), University of Oslo (Norway), and University of Barcelona (Spain). Overall, it reveals a diverse geographical involvement, with institutions from Europe, China, and the United States dominating the academic output (Figure 2D).

### 3.3 Dual‐Map Overlay of Journals

**Figure 3:**
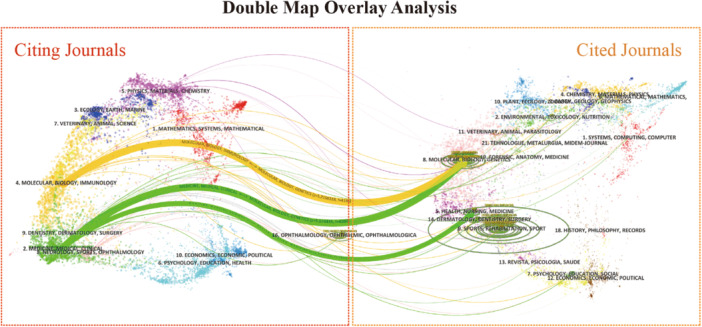
The dual‐map overlay of academic journals in biomarkers of SS. The citing journals are on the left side, while the other side of the map represents the cited journals.

The dual‐map overlay analysis of journals visualizes the interdisciplinary flow of research by linking citing journals on the left with cited journals on the right, providing a comprehensive depiction of knowledge exchange. The analysis reveals three primary pathways: the orange pathway indicates that articles published in *Molecular Biology and Genetics* journals are frequently cited by papers in the *Molecular Biology* and *Immunology* fields. A green pathway highlights the frequent citation of articles from *Molecular Biology and Genetics* journals and *Health, Nursing, and Medicine* journals by papers in *Medicine, Medical and Clinical* journals. Additionally, the size of the ellipses at the edges represents the influence of the cited literature within the respective field. Notably, journals in the *Ophthalmology*, *Ophthalmic*, and *Ophthalmologica* fields also show significant citation frequency, reflecting their substantial impact (Figure 3).

### 3.4 Keyword Analysis

**Figure 4:**
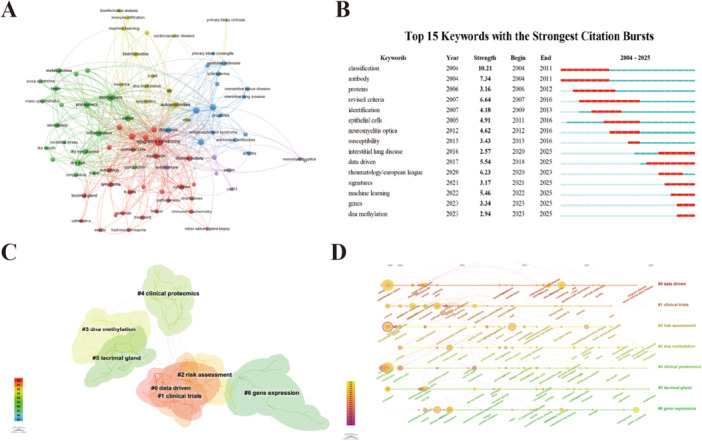
(A) Network visualization of keywords. (B) The top 25 keywords with the most bursts. The years between “Start” and “End” indicate the period when the keyword was markedly affected. The light green color shows that the keyword is still unpublished, dark green indicates a low impact level, while red signifies significant impact. (C) The clustering map of keywords. (D) Timeline distribution of cluster analysis of keywords.

Keywords encapsulate the essence of the literature, and systematic keyword analysis can unveil the evolving trends and research hotspots in SS biomarker research. Keywords appearing more than 6 times were selected for co‐occurrence visualization, where node size represents the frequency of keyword occurrence and edge thickness indicates the degree of co‐occurrence (Figure 4A). A total of 85 entries were identified and grouped into five clusters. The red nodes represent related immunopathological mechanisms and clinical evaluation, including *interferon*, *chemokines*, *essdai*, and *rituximab*. The green nodes denote multi‐omics biomarkers and exocrine gland function such as *metabolomics*, *proteomic*, *saliva*, and *tears*. The blue nodes represent related autoimmune diseases and complications, including *connective tissue disease*, *systemic sclerosis*, *interstitial lung disease*. The yellow nodes highlight epigenetics and bioinformatics, such as *dna methylation*, *microRNA*, *machine learning*. The purple nodes correspond to central nervous system involvement, including *neuromyelitis optica* and *CXCL13*. The multi‐point convergence of the clinical evaluation (Red cluster) and the omics technologies (Green cluster) indicates an active research trend toward establishing non‐invasive, high‐precision molecular diagnostics (Figure 4A). Moreover, among the top 15 emerging keywords, “classification” emerged with the strongest burst strength, indicating that optimizing disease classification standards remains a continuous core issue in this field. The presence of other high‐burst terms like *revised criteria* and *antibody* further suggests a long‐standing focus on establishing precise diagnostic frameworks. Recent emerging keywords such as “machine learning”, “dna methylation”, and “genes” highlight the growing interest in multi‐omics integration, AI model development and epigenetic regulation mechanisms (Figure 4B).

Further analysis using CiteSpace provided more detailed clustering (Figure 4C), identifying six major research domains in SS biomarkers: Cluster 0: Data Driven, which reflects the recent trend of using bioinformatics tools, such as big data and machine learning, to screen for biomarkers; Cluster 1: Clinical Trials, which underscores the importance of biomarkers in assessing drug efficacy, predicting therapeutic response, or monitoring disease activity [10]; Cluster 2: Risk Assessment, which explores how biomarkers predict disease progression, such as the risk of lymphoma transformation and extra‐glandular involvement like interstitial lung disease [11]; Cluster 3: DNA Methylation, which highlights the contribution of epigenetic mechanisms to the development of SS; Cluster 4: Clinical Proteomics, which directly identifies and screening for differentially expressed proteins, autoantibodies, or post‐translational modifications (PTMs) at the protein level; Cluster 5: Lacrimal Gland, a major target of SS, studies on its pathology and tear components represent a hallmark of specificity in this research area; and Cluster 6: Gene Expression, which involves transcriptomic research to identify differentially expressed genes as potential biomarkers [12]. The evolution of these clusters over time is effectively visualized using a timeline, providing deeper insights into the temporal progression of specific research topics (Figure 4D). The evolution trends of these clusters can be divided into three stages: (2004–2010), focusing on the pathological features of salivary and lacrimal glands and the establishment of conventional antibodies; (2012–2018), with the extensive application of proteomic technologies, integrated with clinical validation; and (2019–2025), dominated by epigenetics (Cluster 3), multi‐omics integration (Cluster 0), and risk warning for complications (Cluster 2).

### 3.5 National, Institutional, and Author Network Analysis

**Figure 5:**
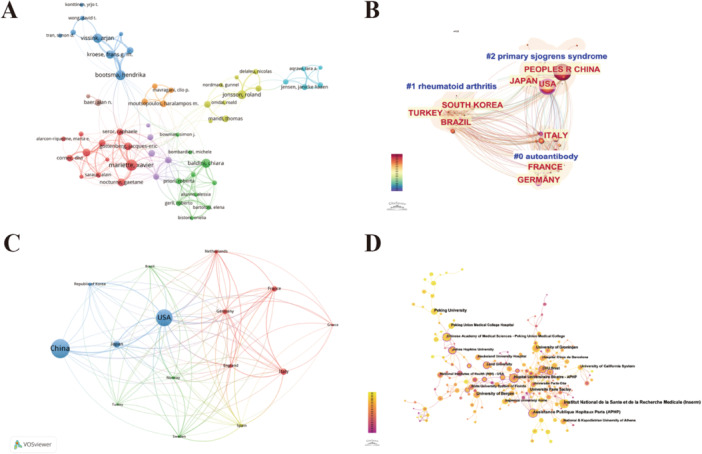
(A) Visualization map of the authors' collaboration network. (B) Thematic clustering of country distribution. (C) Visualization map of countries/regions' collaboration network. (D) Visualization map of institutions' collaboration network.

The author collaboration network reveals that Xavier Mariette, Baldini Chiara, Bootsma Hendrika, and Jonsson Roland are key figures at the core of collaboration clusters, closely linking authors within their respective clusters while also engaging in collaborative ties with one another (Figure 5A). Additionally, we identified the top 10 authors based on publication volume, with Xavier Mariette leading both in terms of publication count and h‐index, positioning him as a central researcher in the field. Following closely, Salvatore De Vita had the highest AC value (89.55), indicating substantial academic influence (Table 1). Geographic differentiation in research topics was observed, with clear regional specialization in SS biomarker studies. Countries such as China, the United States, and Japan dominate the cluster focused on basic and clinical research of this disease, centering on core issues such as the pathogenesis, diagnostic criteria, and clinical phenotyping of SS. A second cluster led by the Republic of Korea, Turkey, and Brazil, emphasizes the intersection of immunology, particularly comorbidity studies between SS and other autoimmune diseases or differential proteomics. Another cluster, led by Italy, France, and Germany is likely concentrated on traditional anti‐SSA/Ro and anti‐SSB/La antibodies, as well as the search for novel biomarkers with high sensitivity and specificity (Figure 5B).

**Table 1:** The top 10 most productive authors contributed to biomarkers of Sjögren's syndrome.

Rank	Author	Country	Affiliations	NP	H‐index	TC	AC
1	Mariette Xavier	France	Universite Paris Saclay	28	21	2243	80.11
2	Baldini Chiara	Italy	University of Pisa	23	19	1371	59.61
3	Bootsma Hendrika	Netherlands	University of Groningen	23	17	982	42.7
4	Jonsson Roland	Norway	University of Bergen	23	18	1420	61.75
5	Gottenberg Jacques‐Eric	France	CHU Strasbourg	20	17	1625	81.25
6	Ramos‐Casals Manuel	Spain	Hospital Clinic de Barcelona	18	14	1511	83.94
7	Salvatore De Vita	Italy	University of Udine	16	9	1433	89.55
8	Vissink Arjan	Netherlands	University of Groningen	16	14	1107	69.19
9	Pers, Jacques‐Olivier	France	Universite de Bretagne Occidentale	15	13	821	54.73
10	Brito‐Zeron Pilar	Spain	Hospital Clinico Universitario	14	11	872	62.29

We also analyzed the collaboration map of the top 13 publishing countries. The United States is the global hub of collaboration, playing a central role in resource integration and knowledge dissemination. Although China shows the largest publication node, its international connectivity is noticeably weaker than that of the United States (Figure 5C). Additionally, we created an institutional collaboration network, where node size represents the institution's participation and centrality in the network—larger nodes indicate closer collaboration. INSERM, AP‐HP, and Johns Hopkins University are core institutions with the most extensive collaboration networks. The Chinese Academy of Medical Sciences stands as the central institution for collaboration in the Asia‐Pacific region, and enhancing cross‐regional cooperation with other international institutions could further bolster its academic influence **(**Figure 5D **)**.

### 3.6 MCA and Co‐Citation Network Analysis of Authors, Journals, and Literature

**Figure 6:**
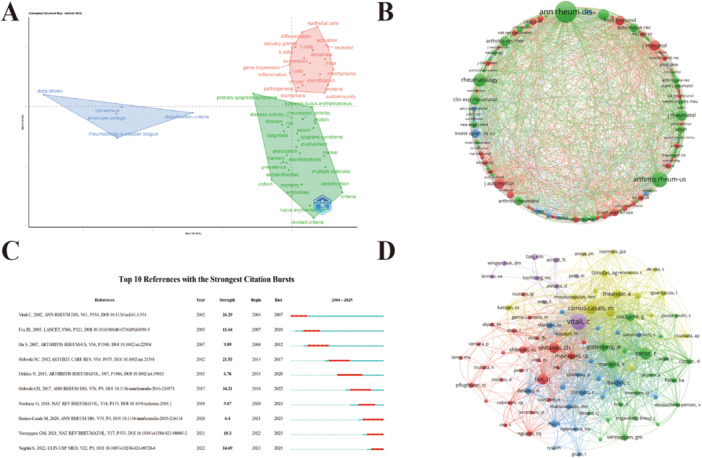
(A) Multiple Correspondence Analysis Chart. (B) Visualization map of the journal co‐citation analysis. (C) The top 10 references with the strongest citation bursts. (D) Visualization map of author co‐citation network.

MCA analysis revealed three primary domains in SS research: diagnostic criteria, biomarker mechanisms, and clinical consensus. Specifically, the Dim 1 axis (51.81%), which predominates the diagnostic‐mechanism dichotomy, reflects the evolving trend of moving from phenotypic definitions to molecular mechanisms. The Dim 2 axis (18.4%), which is independently clustered under clinical consensus, emphasizes the role of standardized practices in driving integrated research. This structure highlights the academic focus of the field and the intersection of clinical and basic research (Figure 6A).

Based on the results of the MCA analysis, the co‐citation networks of authors, journals, and literature were visualized. In the journal co‐citation network, *Annals of the Rheumatic Diseases* emerged as the core journal of the field, with the largest node and the strongest co‐citation relationships, representing the academic center in rheumatology and immunology. *Arthritis & Rheumatology* and *Rheumatology* are also key journals within the rheumatology cluster. The basic immunology cluster includes journals such as *Frontiers in Immunology*, *Journal of Immunology*, and *Clinical and Experimental Rheumatology*. Additionally, specialized journals like *Oral Disease*, *Ocular Disease*, and *Investigative Ophthalmology & Visual Science* show strong co‐citation relationships with the aforementioned journals, highlighting the significance of tears and saliva in SS biomarker research and further revealing the research hotspots in the field (Figure 6B).

In terms of burst analysis, the top 10 most emergent papers reveal the evolution and cutting‐edge dynamics of the field. The paper with the longest burst period (2015–2020) is by Nicolas Delaleu, published in 2015, titled “High Fidelity Between Saliva Proteomics and the Biologic State of Salivary Glands Defines Biomarker Signatures for Primary Sjögren's Syndrome” [13]. This article, using proteomics, identified biomarker combinations and emphasized the advantages of saliva as an ideal sample source—non‐invasive, easily accessible, and directly reflective of the affected organ's state. The paper with the highest burst strength was published in 2017, “2016 American College of Rheumatology/European League Against Rheumatism classification criteria for primary Sjögren's syndrome” [3], which provided unified clinical practice guidelines for SS diagnosis and attracted widespread attention in the U.S. academic community, offering a solid baseline for patient inclusion and definition in SS research. The most recent emergent article is “Sjögren's syndrome: a systemic autoimmune disease” by Simone Negrini et al., published in 2022 [14]. This study not only reflects the high level of attention in academia towards the systemic involvement of Sjögren's syndrome, but also signifies a shift in biomarker research from a single diagnostic function towards multidimensional systemic assessment and precision medicine. These papers demonstrate multidimensional progress in SS research, from basic mechanistic exploration to clinical applications, showcasing the vibrant, interdisciplinary nature of the field (Figure 6C).

In the author network, clusters led by Caroline Shiboski are focused on the development of diagnostic criteria, those led by Baldini Chiara concentrate on biomarker mechanisms, and clusters led by Raphaèle Seror and Xavier Mariette emphasize the clinical applications and translational research of biomarkers. This distribution reflects the leading academic figures in SS research and their contributions to various directions within the field (Figure 6D).

### 3.7 Analysis of Topic Evolution Trends

**Figure 7:**
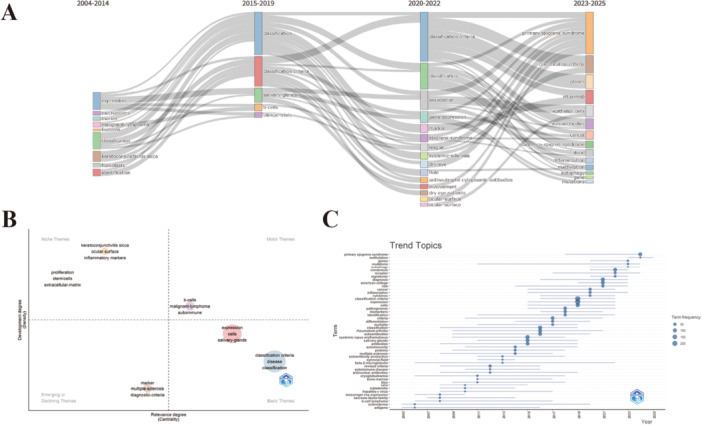
(A) Thematic evolution convergence map. (B) Theme centrality map. (C) Trend Topics.

Over the past two decades (2004–2025), SS biomarker research has exhibited a clear and phased evolutionary trajectory (Figure 7A). Early research (2004–2014) primarily focused on the fundamental definitions of the disease. Core keywords during this period included *classification*, *identification*, and *keratoconjunctivitis sicca*. These studies laid a solid clinical and epidemiological foundation for subsequent in‐depth exploration. The topic centrality map reflects the relative influence of various themes within the research landscape, with *Motor Themes* exhibiting both high centrality and high development potential, such as *malignant‐lymphoma*, *b‐cells*, and *autoimmune*. *Classification criteria* and *salivary‐glands* are identified as the foundational themes supporting the entire field. Conversely, *Niche Themes* are characterized by high density but low centrality. This cluster includes topics such as *ocular surface* and *stem cells*, indicating that research on ocular surface damage and its repair currently constitutes a highly specialized field. In contrast, *Emerging or Declining Themes* feature both low centrality and low density; these may represent topics that are either in their infancy or experiencing a decline in academic attention, such as *multiple sclerosis* and *diagnostic criteria* (Figure 7B).

The topic trend map indicates that during this period, there was considerable focus on these foundational themes. In the mid‐phase of research (2015–2019), the focus was on the development of diagnostic standards, and cellular mechanisms, demonstrating significant theme aggregation and deepening exploration. Core keywords during this phase included *B cell*, *clinical‐trials*, and *classification criteria*. The topic trend map in panel C illustrates the sustained rise in the attention given to these themes. Recent research (2020–2025) has shown a marked clinical translation and precision medicine trend. On one hand, there is an increasing focus on the clinical characteristics of the disease, with core keywords such as *ocular surface*. On the other hand, research has delved deeper into molecular mechanisms, seeking biomarkers with potential diagnostic, prognostic, or therapeutic value. Emerging hotspots include keywords such as *gene* and *methylation*, signaling the rise of molecular‐level exploration as a new focal point (Figure 7C).

## 4 Discussion

This study employed CiteSpace, VOSviewer, and the R package *bibliometrix* to conduct a visualized analysis of the literature related to SS biomarkers, aiming to explore global research trends and key findings. Recent years have witnessed a significant growth in SS biomarker research. As the demand for personalized treatment in SS increases, academic interest in this field has also escalated. This reflects the growing recognition of the importance of biomarkers in SS research, with studies being conducted globally. The geographical analysis of current research reveals a tiered distribution across countries, with China and the United States dominating the number of publications and leading global research directions, while other countries focus on specific areas, creating distinct regional research specialties. At the institutional level, France's AP‐HP stands out, taking a leading position globally. Xavier Mariette is identified as a core researcher in this field, and the author collaboration network demonstrates high levels of cooperation. Scholars from various research directions have formed multiple authoritative clusters, with Xavier Mariette, Baldini Chiara, Bootsma Hendrika, and Jonsson Roland promoting cross‐disciplinary exchanges and advancing research in different domains. *Annals of the Rheumatic Diseases* is identified as the core journal in this field, with an increasing frequency of co‐citations from specialized journals, highlighting significant interdisciplinary collaboration opportunities and development potential in SS biomarker research.

### 4.1 The Evolution of Research Paradigms in SS Biomarkers

By analyzing the most cited articles within the field, we can effectively reveal the inherent connections among the literature and the development trends of the research area. Papers with high cited frequency are often regarded as seminal works or reflective of the most dynamic and active research directions within the field (Table 2). The analysis indicates that early research, represented by studies from Shen Hu and O. H. Ryu, focused on screening SS‐specific salivary proteomic and genomic biomarkers in large cohorts using omics technologies, establishing saliva as a primary biospecimen [15, 16]. Mid‑term research shifted attention toward predicting complications, especially the risk assessment of lymphoma. For instance, articles by Theander and Quartuccio highlighted the value of labial gland histopathology and specific serological markers in lymphoma prediction [17, 18]. Recent studies have turned to refined subtyping and mechanistic investigations. The work of Nezos on interferon signatures integrates biomarkers with the immunological pathogenesis of the disease, specifically linking them to Type I/II IFN signatures [19].

**Table 2:** The top 10 most local cited documents on biomarkers of Sjögren's syndrome.

Rank	Article Title	Author	Journal	Year	Citations
1	Salivary proteomic and genomic biomarkers for primary Sjögren's syndrome	Shen Hu	Arthritis & Rheumatology	2007	53
2	Lymphoid organization in labial salivary gland biopsies is a possible predictor for the development of malignant lymphoma in primary Sjögren's syndrome	Elke Theander	Annals of the Rheumatic Diseases	2011	41
3	Identification of parotid salivary biomarkers in Sjögren's syndrome by surface‐enhanced laser desorption/ionization time‐of‐flight mass spectrometry and two‐dimensional difference gel electrophoresis	O. H. Ryu	Rheumatology	2006	37
4	Serum Levels of Beta2‐Microglobulin and Free Light Chains of Immunoglobulins Are Associated with Systemic Disease Activity in Primary Sjögren's Syndrome. Data at Enrollment in the Prospective ASSESS Cohort	Jacques‐Eric Gottenberg	PLOS ONE	2013	34
5	Proteomic analysis of saliva: a unique tool to distinguish primary Sjögren's syndrome from secondary Sjögren's syndrome and other sicca syndromes	Chiara Baldini	Arthritis Research & Therapy	2011	31
6	Identification of potential saliva and tear biomarkers in primary Sjögren's syndrome, utilizing the extraction of extracellular vesicles and proteomics analysis	Lara A. Aqrawi	Arthritis Research & Therapy	2017	31
7	Preclinical Validation of Salivary Biomarkers for Primary Sjögren's Syndrome	Shen Hu	Arthritis Care & Research	2010	25
8	Biomarkers of lymphoma in Sjögren's syndrome and evaluation of the lymphoma risk in prelymphomatous conditions: Results of a multicenter study	Luca Quartuccio	Journal of Autoimmunity	2014	25
9	Type I and II interferon signatures in Sjogren's syndrome pathogenesis: Contributions in distinct clinical phenotypes and Sjogren's related lymphomagenesis	Adrianos Nezos	Journal of Autoimmunity	2015	24
10	Proteome analysis of whole saliva: A new tool for rheumatic diseases – the example of Sjögren's syndrome	Shen Hu	Arthritis & Rheumatology	2007	53

The burst detection analysis (Figure 4C), timeline distribution of keyword cluster analysis (Figure 4D), and trend topics (Figure 7C) reveal a systematic shift in the research landscape. Overall, research on biomarkers of SS exhibits two primary developmental trends: (1) a functional shift from auxiliary diagnosis to risk prediction, and (2) a paradigm shift from traditional autoantibody detection toward multi‐omics integration.

In terms of functional positioning, the focus of biomarker research is transitioning from auxiliary diagnosis to early‐warning assessment. During the early stage (2004–2011), research was highly concentrated on the standardization of diagnostic criteria and the identification of fundamental antibodies. The frequent emergence of burst terms such as *classification*, *antibody*, and *identification reflects* the urgent clinical need to establish unified disease classification standards. In the middle stage (2012–2016), with the emergence of terms like *neuromyelitis optica* and *susceptibility*, research began to expand into organ‐specific involvement and genetic predisposition [20]. More recently, trend topics from 2020 to 2025 show a significant increase in node sizes for *interstitial lung disease*, *risk*, and *mortality*. Coupled with updates to the *Rheumatology/European League* prognostic standards, this provides strong evidence that the current research focus has shifted toward early warning and individualized risk assessment for complications such as interstitial lung disease and lymphoma.

In terms of research paradigms, biomarkers are evolving from traditional autoantibody detection toward multi‐omics integration. Before 2010, research remained primarily at the traditional antibody detection stage, centered on anti‐SSA/Ro and anti‐SSB/La antibodies. These autoantibodies were mainly detected via immunological methods such as ELISA, with research focusing on their correlation with clinical phenotypes. However, because approximately one‐third of patients are seronegative, traditional antibody research reached a diagnostic bottleneck. Subsequently, the cluster #4 clinical proteomics and the burst term *proteins* marked a transitional phase into serum protein detection. Since 2020, with the maturation of high‐throughput technologies, the research paradigm has formally entered a multi‐dimensional integration phase. On one hand, transcriptomic research has matured, with *expression* and *signatures* becoming high‐frequency core terms. On the other hand, epigenetics has emerged as the latest growth point; *DNA methylation*, as the most recent burst term for 2023–2025, reveals a deeper exploration of regulatory mechanisms. Furthermore, the frequent appearance of *data‐driven* and *machine learning* provides the necessary technical support for processing complex multi‐omics integrated data. This suggests that, within the context of precision medicine, SS biomarkers are now being utilized to investigate disease heterogeneity from a holistic perspective.

### 4.2 The Future Hotspots and Frontiers in SS Biomarkers

Based on burst keyword detection, emerging topics such as machine learning, *DNA methylation*, *genes*, and *interstitial lung disease* have demonstrated sustained growth signals. The timeline evolution further reveals that the research cluster centered on data‐driven approaches (Cluster 0) has expanded markedly in recent years. Similarly, within the gene expression domain (Cluster 6), directions like microRNA and circRNA continue to emerge and develop rapidly. Through integrating keyword burst dynamics and thematic evolution trends, the present study highlights three key areas shaping current priorities and future research directions in SS biomarker studies:

1. Epigenetic biomarkers (DNA methylation, miRNA, circRNA);
2. Complication prediction biomarkers (related to lymphoma and interstitial lung disease);
3. Multi‐omics integration and technological innovations.

DNA methylation, a crucial epigenetic modification, plays a key role in the pathogenesis of SS. In the DNA methylation process, DNA methyltransferases (DNMTs) transfer methyl groups from S‐adenosylmethionine (SAM) to cytosine‐guanine (CpG) dinucleotides, resulting in 5‐methylcytosine (5mC) at the carbon‐5 position of the cytosine ring [21]. CpG methylation leads to chromatin structure alterations, hindering transcription factor binding. Studies have shown that various cell types in SS, such as T cells, B cells, and salivary gland epithelial cells, exhibit DNA methylation defects [22, 23, 24]. In gene promoter regions, hypermethylation at CpG sites typically suppresses gene expression, while hypomethylation may be associated with increased gene expression levels. Compared to other epigenetic changes, DNA methylation offers greater stability, making it a promising diagnostic biomarker [25]. For instance, whole‐genome methylation sequencing revealed that the high expression of IFN‐regulated IFI44L gene is significantly correlated with disease activity scores and clinical indices in SS patients, with its DNA methylation status potentially serving as a valuable biomarker for assessing disease activity [26].

miRNAs, endogenous non‐coding small RNA molecules, play critical roles in various biological processes, including cell growth, differentiation, and apoptosis [27]. Due to their stability and circulation in body fluids such as saliva and tears, as well as their association with SS onset and progression, miRNAs have become a research hotspot in SS biomarker studies [28]. Yamashiro et al. identified significantly elevated miR‐1290 and let‐7b‐5p in the exosomes from oral rinse of SS patients, and these miRNAs, when combined, can accurately distinguish SS patients from healthy controls with high sensitivity and specificity [29]. Other studies have also identified differentially expressed miRNAs in tears from mice and humans [30, 31]. These miRNAs hold potential as non‐invasive biomarkers for SS diagnosis and disease monitoring. However, there remain challenges in miRNA research in SS, including discrepancies in reported diagnostic or prognostic miRNAs across studies, which may be attributed to sample sources, detection methods, and disease heterogeneity [32]. Furthermore, identifying clinically relevant miRNAs from numerous differentially expressed miRNAs and standardizing miRNA detection techniques to enhance accuracy and reproducibility remain unresolved issues. To optimize miRNA research in SS biomarker development, further studies with larger, multi‐center cohorts are necessary, alongside standardized sample collection, handling, and detection protocols, to better understand miRNA's role in SS pathogenesis and its regulatory networks.

CircRNAs, a special class of non‐coding RNAs with a closed‐loop structure, are more stable than linear RNAs and are less susceptible to degradation by nucleases [33]. Recent studies suggest that circRNAs participate in the immune regulation and pathological processes of SS through mechanisms such as miRNA sponging and protein interactions [34]. Due to their stability and unique biological functions, circRNAs are promising candidates for early SS diagnosis, disease monitoring, and prognostic evaluation [35]. Several differentially expressed circRNAs, such as hsa_circ_0045800, have been identified in the peripheral blood of pSS patients, with a sensitivity of 74% and specificity of 92%, indicating their high potential as diagnostic biomarkers [36]. Additionally, circ‐IQGAP2 and circ‐ZC3H6 were upregulated in exosomes from pSS patients and may serve as non‐invasive biomarkers [10]. However, circRNA research in SS is still in its infancy, with most studies limited to differential expression analysis, and more work is needed to explore their mechanisms and clinical applications. Further research into the molecular regulatory networks of circRNAs in SS pathogenesis is essential, as well as the development of more sensitive and specific detection techniques for circRNA biomarkers.

Lymphoma and interstitial lung disease are common and severe complications in SS patients, significantly increasing disease burden and mortality risk [37, 38]. Identifying biomarkers that can effectively predict and monitor the development of these complications is crucial for improving patient prognosis. Abnormal B cell activation in SS patients can lead to chronic inflammation and lymphocyte proliferation, ultimately resulting in lymphoma. Studies have shown that B cell activating factor (BAFF) and BAFF receptor (BAFF‐R) are upregulated in SS patients and are closely associated with lymphoma development [39]. Moreover, low levels of miR200b‐5p in minor salivary glands may serve as a new molecular biomarker for predicting lymphoma development in SS patients [40]. These findings provide molecular evidence for lymphoma risk stratification and offer directions for targeted therapy. In interstitial lung disease associated with SS, patients often present with progressive dyspnea and dry cough, which significantly affect quality of life and prognosis [41]. Sialoglycan antigen 6 (KL‐6), a high‐molecular‐weight glycoprotein predominantly expressed by type II alveolar epithelial cells and bronchial epithelial cells, is closely linked to lung injury and fibrosis [42]. Several studies have indicated that KL‐6 levels are significantly elevated in the serum of SS‐related ILD patients, with its changes correlating positively with the degree of lung function damage and radiological progression [43, 44]. In addition, in‐depth studies of molecular mechanisms, including proteomics, metabolomics, and genetic biomarkers, are providing new directions for the discovery of biomarkers.

Data‐driven multi‐omics technologies, such as genomics, transcriptomics, proteomics, and metabolomics, offer powerful tools for identifying reliable biomarker combinations for disease subtyping, risk prediction, and therapeutic response modeling [45]. For example, integrating genomic and proteomic data can uncover key genes and the expression changes of their encoded proteins associated with SS, thereby identifying biomarker combinations with diagnostic and prognostic value [46]. A novel diagnostic approach combining ultrasound and transcriptomic biomarkers has achieved diagnostic accuracy for pSS comparable to the gold standard of minor salivary gland biopsy. Additionally, Joe Scott Berry and colleagues used proteomics and network analysis to examine the pathological and biological characteristics of SS subgroups, providing a theoretical foundation for disease subtyping and personalized treatment [47]. However, multi‐omics technologies face challenges in practical applications, such as large data volumes, the complexity of data integration, and intricate interrelationships between different omics data. To fully leverage the advantages of multi‐omics in SS biomarker research, there is a need to further develop and refine bioinformatics methods to enhance data processing and analysis efficiency, foster interdisciplinary collaboration, and enable deep data mining and effective utilization of multi‐omics data.

## 5 Limitations and Deficiencies

This study employed CiteSpace to conduct a visualized analysis of English‐language literature from the Web of Science Core Collection (WoSCC), mapping research hotspots and trends in SS biomarkers. However, the WoSCC's exclusion of regional databases limits the inclusion of high‐quality research published in non‐English languages, potentially overlooking key contributions from non‐English‐speaking countries. Additionally, the absence of gray literature, such as conference papers and preprints, may hinder the tracking of emerging trends and cutting‐edge technologies. Although continuous database updates improve timeliness, they may underrepresent foundational studies, affecting the comprehensive portrayal of the field's development.

## 6 Conclusion

This study is the first systematic bibliometric and visualized analysis of SS biomarker research, offering a comprehensive overview of key countries, institutions, authors, journals, papers, and keywords in the field. It highlights the evolution of research trends, with a clear shift from basic mechanistic exploration prior to 2019 to a focus on clinical applications afterward. Future SS targeted therapies should prioritize the development of stable, non‐invasive biomarkers through liquid biopsy technologies, strengthen clinical research on biomarkers for complications like lymphoma and interstitial lung disease, and leverage multi‐omics and AI‐driven models for better patient stratification and precision treatment. Overall, our research provides valuable insights into the trends and frontiers of SS biomarker research, fostering a deeper understanding and advancing the field.

## Author Contributions

**Xinyan Zhang:** conceptualization, methodology, software, data curation, investigation, validation, formal analysis, visualization, writing – original draft. **Huan Li:** conceptualization, methodology, software, data curation, investigation, validation, formal analysis, visualization, writing – review and editing. **Xueqing Zhou:** software, data curation, visualization, writing – original draft. **Shangwen Qi:** software, data curation, visualization, writing – original draft. **Songwei Li:** supervision, funding acquisition, project administration, resources, writing – review and editing.

## Ethics Statement

The authors have nothing to report.

## Conflicts of Interest

The authors declare no conflicts of interest.

## Data Availability

Data sharing not applicable to this article as no datasets were generated or analysed during the current study. The original contributions presented in the study are included in the article/Supporting material, further inquiries can be directed to the corresponding author.
